# Significant Increase of Cinnamic Acid in Metabolites of Chicks Infected with Infectious Bronchitis Virus and Its Remarkable Antiviral Effects In Vitro and In Vivo

**DOI:** 10.3390/microorganisms13071633

**Published:** 2025-07-10

**Authors:** Lan-Ping Wei, Tao-Ni Zhang, Yu Zhang, Li-Na Ren, Yan-Peng Lu, Tian-Chao Wei, Teng Huang, Jian-Ni Huang, Mei-Lan Mo

**Affiliations:** 1 College of Animal Science and Technology, Guangxi University, Nanning 530004, China; 2 Guangxi Zhuang Autonomous Region Engineering Research Center of Veterinary Biologics, Nanning 530004, China; 3 Guangxi Key Laboratory of Animal Breeding, Disease Control and Prevention, Nanning 530004, China

**Keywords:** infectious bronchitis virus, metabolomics, cinnamic acid, antiviral, N protein

## Abstract

Avian infectious bronchitis virus (IBV) infection has caused significant economic losses to the poultry industry. Unfortunately, there is currently no effective cure for this disease. Understanding the pathogenic mechanism is crucial for the treatment of the disease. Studying the pathogenic mechanism of IBV based on metabolomics analysis is helpful for identifying antiviral drugs. However, studies on metabolomics analysis of IBV infection have been relatively limited, particularly without metabolomics analysis in sera after IBV infection. In this study, 17-day-old SPF chicks were infected with the IBV GX-YL5 strain, and serum samples were collected 7 days post-infection (DPI) for metabolomics analysis using ultraperformance liquid chromatography tandem mass spectrometry (UPLC-MS/MS). A total of 143 differential metabolites were identified across 20 metabolic pathways, with the phenylalanine pathway showing the most significant changes. The level of cinnamic acid (CA), an upstream metabolite in the phenylalanine pathway, was notably increased following IBV infection. To investigate the antiviral effects of CA, chicken embryo kidney (CEK) cells and SPF chicks infected with IBV were treated with different concentrations of CA to assess its effect on viral replication. The results demonstrated that CA at 25 μg/mL effectively inhibited IBV replication in vitro; meanwhile, CA at 50 μg/mL and 25 μg/mL effectively inhibited IBV replication in vivo. Molecular docking and molecular dynamics simulation studies showed that CA interacts with the N domains of the IBV nucleocapsid (N) protein. In conclusion, the serum metabolite CA is significantly elevated following IBV infection and demonstrates remarkable antiviral effects both in vitro and in vivo, providing a promising avenue for the development of antiviral therapies to combat IBV infection.

## 1. Introduction

Avian infectious bronchitis virus (IBV), a member of coronavirus family, causes acute, highly contagious respiratory disease in chickens and is associated with significant economic losses to the poultry industry worldwide [1]. Currently, vaccination remains the most reliable measure for controlling IBV infection. However, the protective efficacy of vaccines is limited due to viral high mutation rates [2,3,4]. At present, there is no effective treatment strategy for IBV. Therefore, in addition to safe and effective vaccines, broad-spectrum antiviral drugs are urgently needed for the prevention and control of IBV.

IBV is a positive-strand RNA virus, and the total length of the viral genome is approximately 27.6 kb [5]. IBV encodes four structural proteins, including the spike (S) protein, nucleocapsid (N) protein, membrane (M) protein, and envelope or small membrane (E) protein. Among them, the N protein is regarded as highly conserved, and it consists of an N-terminal domain (N-NTD) and a C-terminal domain (N-CTD) [6]. The N-NTD is the core region where the N protein binds to RNA and is responsible for directly binding to the viral genomic RNA to form the nucleocapsid [7]. This function is crucial for the packaging and transcription of viral RNA. The N-CTD is another key functional region of the N protein, involved in the formation of N protein dimers [8]. In addition, the N protein is also related to the assembly, germination, and regulation of immune responses to the virus. Due to its key role in the process of viral infection of the host, the N protein has become an attractive target for the development of potential antiviral drugs [9].

A better understanding of the disease process is essential for developing effective strategies to prevent and control diseases. Metabolomics, as a high-throughput platform, enables researchers to capture metabolic alterations that reflect the underlying disease mechanisms and further investigate antiviral drugs [10]. So far, studies on the metabolic changes in IBV infection have been relatively limited. Previous studies demonstrated that metabolic profiling in the kidney and bursa of Fabricius of chickens infected with IBV exhibited significant changes [11,12]. However, data on serum metabolite changes during IBV infection in chickens remain scarce. The metabolic characteristics of serum samples offer distinct advantages, as serum metabolomics data collected at specific times provide insights into “short-term” biomarkers that reflect the current state of host–pathogen interactions [13]. Therefore, it is highly necessary to conduct serum metabolomics research on IBV infection.

In recent years, the study of small molecule compounds has garnered increasing attention, particularly focusing on the diverse physiological effects and functions of cinnamic acid (CA) and its derivatives [14]. CA has a variety of pharmacological properties such as anti-inflammatory, antioxidant, and antiviral [15]. Previous studies have shown that CA inhibits Zika virus and hepatitis C virus replication [16,17]. However, the underlying molecular mechanisms of CA’s antiviral effect remain poorly understood. Whether CA can inhibit IBV replication deserves further investigation.

In this study, we infected chicks with IBV and conducted a metabolomics analysis of serum metabolism in IBV-infected chicks, leading to the identification of a significant increase in metabolite CA. Further investigation revealed that CA could remarkably inhibit IBV replication in vitro, and in vivo CA inhibits IBV replication by targeting the IBV N protein. To our knowledge, this is the first report to demonstrate the antiviral effects of CA against IBV infection based on metabolomics analysis, providing a novel strategy for the prevention and treatment of IBV infection.

## 2. Materials and Methods

### 2.1. Cells, Virus, and CA

Chicken embryo kidney (CEK) cells were maintained in our laboratory and cultured in Dulbecco’s modified Eagle’s medium (DMEM, Procell, Wuhan, China), supplemented with 10% fetal bovine serum (FBS, Procell, Wuhan, China) and 1% penicillin, streptomycin, and gentamicin (Solarbio, Beijing, China) under 5% CO_2_ at 37 °C. The IBV strain GX-YL5 (accession number, HQ 848267.1) was isolated by our research group [18]. CA (≥98.0%) was purchased from Solabao Company (Beijing, China).

### 2.2. Animal Experimental Design

Fifty 17-day-old specific-pathogen-free (SPF) chicks, purchased from Xinxing Dahuanong Poultry Eggs Co., Ltd., Guangzhou, China, were randomly assigned to two groups: 25 chicks in the IBV-infected group and 25 chicks in the control group. The chicks were housed in separate isolation rooms equipped with individual ventilation systems. Chicks in the IBV-infected group were infected oculonasally with 0.2 mL of 10^6^ EID_50_ IBV GX-YL5 allantoic fluid, while those in the control group received 0.2 mL of sterilized negative allantoic fluid under the same conditions. Blood samples were collected aseptically from the wing veins of chicks at 0, 3, 7, 11, and 14 days post-infection (DPI). The blood samples were naturally agglutinated at room temperature for 30–60 min, then the clotted blood was centrifuged at 2000–3000 rpm for 5–10 min to obtain serum samples for the detection of IBV-specific antibody levels. At 7 DPI, six serum samples from each group were collected and sent to Met Ware Co. Ltd. (Dong Hu, Wuhan, China) for metabolite detection using ultra-performance liquid chromatography tandem mass spectrometry (UPLC-MS/MS) (ExionLC AD UPLC-QTRAP, SCIEX, Boston, MA, USA). Following sera collection, five chicks from both the IBV-infected and control groups were euthanized at 0, 3, 7, 11, and 14 DPI to collect kidney and tissue samples for the detection of IBV viral loads. The animal experiments were approved by the Animal Ethics Committee of Guangxi University (GXU-2022-282).

### 2.3. ELISA Detection of IBV-Specific Antibody

The IBV-specific antibody levels were determined at 0, 3, 7, 11, and 14 DPI using an indirect ELISA according to the previous description [19]. The polyclonal antisera, serving as the primary antibody, was serially diluted in phosphate buffer saline (PBS) into eight concentrations: 1:200, 1:400, 1:800, 1:1600, 1:3200, 1:6400, 1:12,800, and 1:25,600. Subsequently, HRP-conjugated goat anti-chicken IgY secondary antibody was diluted 1:5000 in PBS. The optical density (OD) values were read using a microplate reader (iMark; Bio-Rad, Hercules, CA, USA) at a 450 nm wavelength.

### 2.4. UHPLC-ESI-MS/MS Analysis and Metabolite Identification

The hydrophilic and hydrophobic compounds from serum samples were extracted following a previously described protocol [20]. Metabolic profiling and identification of differential metabolites in hydrophilic and hydrophobic samples were performed using the UHPLC-ESI-MS/MS system (UPLC, Shim-pack UFLC SHIMADZU CBM A system, https://sciex.com.cn/, accessed on 6 June 2025; MS, QTRAP^®^ 6500+ System, https://sciex.com/, accessed on 6 June 2025) (SCIEX, Boston, MA, USA) and a proprietary software database, including: R (base package) 3.5.0 and MetaboAnalystR (R) 1.0.1. Quantification of metabolites was achieved through multiple-reaction monitoring using triple quadrupole mass spectrometry [21]. The changes in metabolite content are expressed as the relative differences between the IBV-infected group and the control group. The metabolic pathways involved in differential metabolites were analyzed by the Kyoto Encyclopedia of Genes and Genomes (KEGG) enrichment.

### 2.5. Cytotoxicity Assay of CA

To evaluate the toxic effect of CA on CEK cells, the cells were seeded at a density of 1 × 10^4^ cells/well into 96-well culture plates and incubated at 37 °C in 5% CO_2_ for 24 h. Following incubation, the cell plates were washed three times with PBS. CA was then added at various concentrations (0, 25, 50, 100, 200, 400, 800, and 1600 μg/mL), with three replicates prepared for each concentration. The cells were subsequently incubated with CA for 48 h. Cell viability was assessed using the cell counting kits-8 (CCK8) assay according to the instructions. The absorbance was measured at 450 nm using a Quant universal microplate spectrophotometer (Biotex, Houston, TX, USA). The relative survival rate of the cells was calculated based on a previously described method [22]. When the drug concentration was ≤CC_50_ (semi-maximum inhibitory concentration), it was considered non-toxic [23].

### 2.6. In Vitro Analysis of the Effect of CA on IBV Replication

To investigate the effect of CA on IBV replication in vitro, CEK cells were treated with various concentrations of CA (drug group: 0, 6.25, 12.5, and 25 μg/mL) and infected with IBV at a multiplicity of infection (MOI) of 1.0. IBV-infected cells were used as the IBV-infected group, and uninfected CEK cells were used as the control group. After 24 h of CA treatment, the supernatant from CEK cells was harvested into a 2.5 mL sterile tube using a pipette, filtered through a 0.22 μm sterile filter, and designated as the viral stock. This stock was serially diluted in a 10-fold manner, ranging from 10^−1^ to 10^−8^. The diluted viral solutions were then used to infect CEK cells in a 96-well cell plate with 100 μL per well, and the infectivity of the viral fluid was assessed using cytopathic effect (CPE) analysis [24]. Additionally, cell sediment was collected to assess viral loads via real-time quantitative PCR (qPCR) [25] and measure the content of IBV-N protein using western blotting. The rabbit polyclonal IBV N protein antibody (prepared in our laboratory, 1:2000 dilution) and mouse monoclonal anti-GAPDH antibody (Abmart, Shanghai, China, 1:5000 dilution) were used as the primary antibodies. The signal was further detected by HRP-labeled goat anti-rabbit IgY or goat anti-mouse IgY secondary antibodies (Abmart, Shanghai, China; diluted 1:5000).

### 2.7. In Vivo Analysis of the Effect of CA on IBV Replication

Seventy-five 14-day-old SPF chicks were randomly assigned into 5 groups, each consisting of 15 chicks: the blank control, CA-0, CA-10, CA-25, and CA-50 groups (Table 1). With the exception of the blank control group, the chicks in the other groups were infected oculonasally with 0.2 mL of 10^5^ EID_50_ IBV GX-YL5 allantoic fluid. Each treatment group received the prescribed dose of the drug via drinking water for five consecutive days. On the second day after drug withdrawal, five chickens from each group were euthanized, and tracheal and kidney tissues were collected for viral load detection and histopathological examination.

### 2.8. Detection of Virus Loads by qPCR

qPCR was performed to evaluate the mRNA levels in kidney and tracheal tissues using the SYBRs Premix Ex Taq™ kit (Vazyme, Nanjing, China), as previously described [18]. Absolute quantification was conducted to determine the absolute copy numbers of IBV-N mRNA based on the standard curve derived from the cycle threshold (Ct) values [25].

### 2.9. Histopathological Examination

On the second day after drug withdrawal, the trachea and kidneys were collected and immersed in 10% neutral formalin fixative solution for fixation for more than 48 h, with a fixative to tissue ratio of 10:1. The fixed tissue blocks were then dehydrated, embedded in paraffin, and processed into thin sections using a microtome. These sections were stained with hematoxylin-eosin and examined under an optical microscope for histopathological evaluation.

### 2.10. Molecular Docking of CA and Protein

The 2D structure of CA (Compound CID: 444539) was downloaded from the PubChem database (https://pubchem.ncbi.nlm.nih.gov/, accessed on 6 July 2025) and saved as an “sdf” format file. The small molecule library (ligands) was established by using the “New Database” function of the Molecular Operating Environment (MOE2019) software [26]. The 3D structure of NTD (PDB: 2BTL) and CTD (PDB: 2GE7) protein of IBV N were downloaded from the PDB database (https://www.rcsb.org, accessed on 6 June 2025). After minimizing the energy of the small ligands and proteins, molecular docking of the small ligands and proteins was performed using the MOE software. The docking results were visualized and analyzed to obtain the docking complex of protein–small molecule compounds.

### 2.11. Molecular Dynamics Simulation

Molecular dynamics simulation (MD) was conducted using the Gromacs2022 program [27]. For small molecules, the GAFF force field was used [28]. For proteins, the AMBER14SB force field and the TIP3P (transferable intermolecular potential 3 points) water model were adopted [29,30]. We combined the files of proteins and small molecule ligands to construct a simulation system of the complex. The system construction details were as follows: PH: 7.4 (His residues neutral), box type: regular 16hedral, box padding: 1.2 nm, water molecules: 12,348 ± 2.5 (system-dependent), and neutralization: 0.15 M NaCl + counterions (Na^+^/Cl^−^). It was performed under constant temperature and pressure, as well as periodic boundary conditions. During the MD simulation process, all hydrogen bonds involved were constrained by the LINCS algorithm [31], and the integration step size was 2fs. The electrostatic interaction was calculated by the particle-mesh Ewald (PME) method [32], and the cut-off value was set at 1.2 nm. The cut-off value of non-bond interaction was set to 1.0 nm and updated every 10 steps. The simulated temperature was controlled at 298 K by the V-rescale temperature coupling method, and the pressure was controlled at 1 bar by the Berendsen method. Production MD: NTD-CA complex: 100 ns (3 independent replicates), CTD-CA complex: 100 ns (3 independent replicates). After the simulation was completed, VMD and pymol were used to analyze the simulated trajectories [33,34], and the g_mmpbsa binding free energy between proteins and small molecule ligands was analyzed using the g_mmpbsa program [35]. The stability and convergence of small molecule and protein binding were judged by analyzing RMSD, distance, and the number of hydrogen bonds between CA and the active pocket of the protein [36].

### 2.12. Statistical Analysis

Data are presented as mean ± standard error of the mean (SEM). Statistical analyses were performed using GraphPad Prism 5 (GraphPad Software, La Jolla, CA, USA). One-way analysis of variance (ANOVA), followed by the Duncan test, was performed to compare differences among multiple groups. *p* values < 0.05 were considered statistically significant.

## 3. Results

### 3.1. The Dynamics of Viral Loads and Antibody Levels in IBV-Infected Chicks

**Figure 1:**
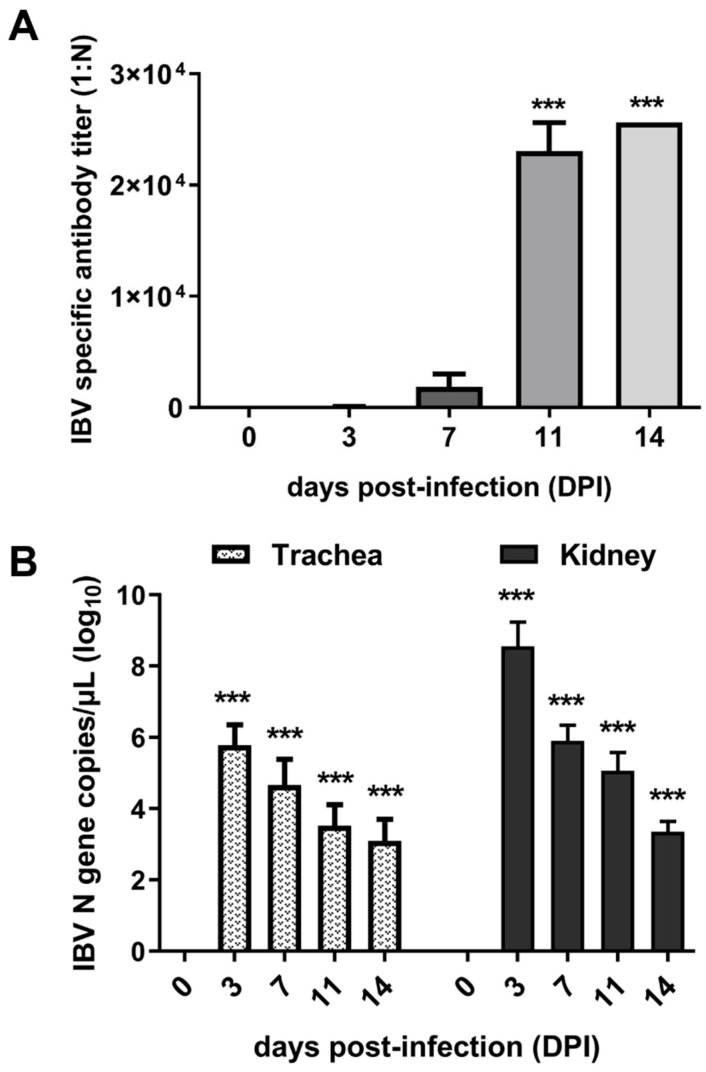
Expression of viral load and antibody levels. (**A**) Titers of anti-IBV antibody in sera of infected chicks. (**B**) Viral loads in the trachea and kidney of chicks. *** indicates *p* < 0.001.

To confirm the successful infection of IBV, antibody levels were determined using ELISA, and viral loads in the trachea and kidney of infected chicks were detected using qPCR. The results demonstrated that the antibody levels gradually rose over time following infection. Notably, serum antibody titers exhibited a sharp increase between 7 and 11 DPI (Figure 1A). Viral load analysis revealed that the virus maintained high levels of proliferation at 3 and 7 DPI, followed by a significant decrease at 11 and 14 DPI (Figure 1B). Collectively, the dynamics of the antibodies and viral loads demonstrated successful infection of IBV in chicks. Furthermore, the observed antibody levels suggested that 7 DPI may represent a critical time point for studying the interaction between IBV and hosts.

### 3.2. Multivariate Statistical Analysis of Metabolites

**Figure 2:**
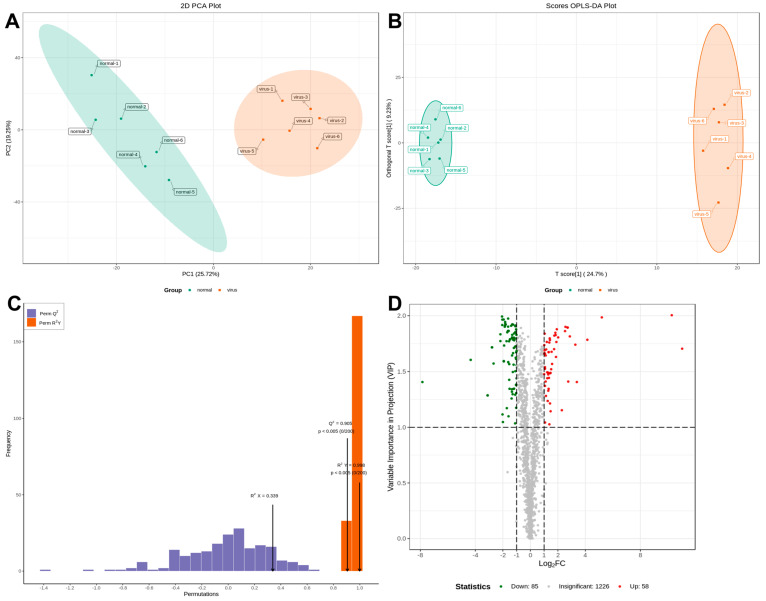
Plots for multivariate statistical analysis and metabolite analysis. (**A**) PCA scores. (**B**) PLSDA score plot. (**C**) OPLS-DA score plot. The R2X (cum) and R2Y (cum) are 0.339 and 0.998, respectively. Q2 is 0.905. (**D**) Volcano maps were used to show all metabolites detected. Green dots represent downregulated differentially expressed metabolites, red dots represent upregulated differentially expressed metabolites, and gray dots represent unchanged metabolites.

Serum samples from IBV-infected and control chicks were analyzed using non-targeted HPLC-MS/MS metabolomics. Multivariate statistical analysis was conducted to assess the reliability of the obtained sample data. The principal component analysis (PCA) results revealed distinct clusters between the two groups, indicating significant differences in metabolite profiles (Figure 2A). The partial least squares discriminant analysis (PLS-DA) and orthogonal partial least squares discriminant analysis (OPLS-DA) models exhibited high performance, with R2Y = 0.998 and Q2 = 0.905 (Figure 2B,C). These values, approaching 1, demonstrated excellent model fitting and predictive capabilities. The findings revealed significant alterations in sera metabolites following IBV infection. Differential sera metabolites were identified using the OPLS-DA model and normalized peak area comparisons. Metabolites were screened based on stringent criteria: *p*-value < 0.05 and VIP > 1. From a total of 1369 metabolites, 143 were identified as differentially expressed. Among these, 58 were upregulated, while 85 were downregulated in the sera of IBV-infected chicks (Figure 2D).

### 3.3. Metabolic Pathway Enrichment Analysis

**Figure 3:**
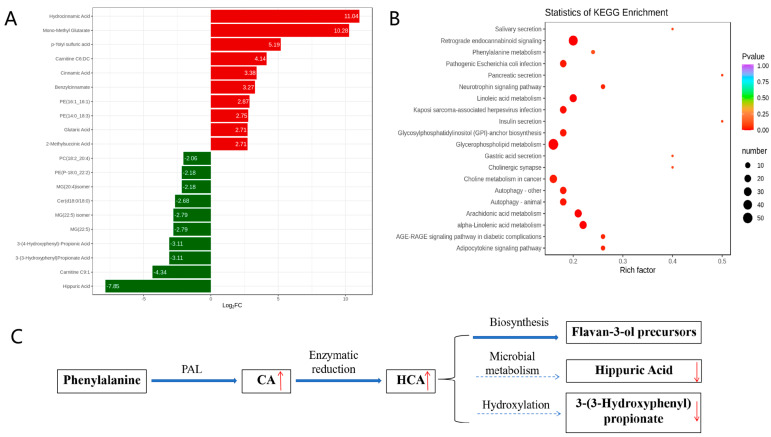
Analysis of metabolic pathways involved in metabolites. (**A**) Differentially expressed metabolites. (**B**) KEGG enrichment analysis of significant differential metabolites. (**C**) Rewiring of phenylalanine metabolism during IBV infection. In (**A**), the horizontal axis represents the logarithm of the difference multiple, and the vertical axis represents the metabolites. Red indicates up-regulation and green indicates down-regulation. In (**B**), the color of the point represents the *p*-value; the redder it is, the more significant the enrichment. The size of the dots represents the number of differential metabolites enriched. In (**C**), Regulation direction: upward (red upward arrow) and downward (red downward arrow). Metabolic flow: main metabolic flow (solid arrow) and inhibited metabolic flow (dashed arrow).

KEGG enrichment analysis of the metabolic pathways was conducted to investigate the mechanism of metabolic pathway changes after IBV infection. A fold change (FC) ≥ 2.0 was used as the cutoff for identifying significant differences. The volcano plot analysis (Figure 3A) identified 143 differential metabolites, with 58 upregulated (including CA and HCA) and 85 downregulated (including hippuric acid and 3-(3-hydroxyphenyl)propionate)**.** This supports host-driven redirection of phenylalanine catabolism away from microbial degradation toward defense metabolite production. As detailed in Figure 3B, these alterations map to the phenylalanine metabolic pathway: CA is synthesized from phenylalanine via phenylalanine ammonia-lyase (PAL), HCA is generated by enzymatic reduction of CA, HCA channels flux into flavan-3-ol biosynthesis (KEGG map00941), and downregulation of catabolites indicates metabolic diversion from degradation. Furthermore, KEGG enrichment confirmed broad metabolic reprogramming, with 20 pathways significantly enriched (Figure 3C). The phenylalanine pathway showed the most pronounced changes, indicating rewired metabolic activity during infection.

### 3.4. Cytotoxic Effect of CA on CEK Cell Proliferation

**Figure 4:**
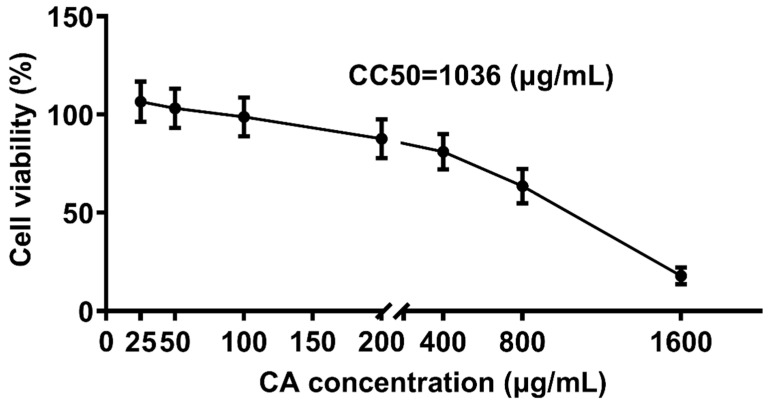
Cytotoxic effect of CA treatment on CEK cells. CEK cells were treated with 0, 25, 50, 100, 200, 400, 800, or 1600 μg/mL of CA for 48 h. Values were normalized to the value of the control group (set at 100%).

To determine the non-toxic concentrations of CA, CEK cells incubated with different concentrations of CA (25, 50, 100, 200, 400, 800, and 1600 μg/mL) for 48 h were evaluated for cell viability by CCK8 assays, and normal cells (0 μg/mL) were set as the control group (Figure 4). The corresponding cell viability percentages of CA-treated groups were 108%, 103%, 97%, 86%, 74%, 62%, and 18%, respectively. The results indicated that CA at concentrations of 25 and 50 μg/mL promoted cell growth, whereas CA at 1600 μg/mL reduced cell viability by over 50%. The calculated CC50 for CA was 1036 μg/mL. Therefore, concentrations lower ≤ 1036 μg/mL were considered non-toxic and selected for subsequent experiments.

### 3.5. CA Inhibits IBV Replication In Vitro

**Figure 5:**
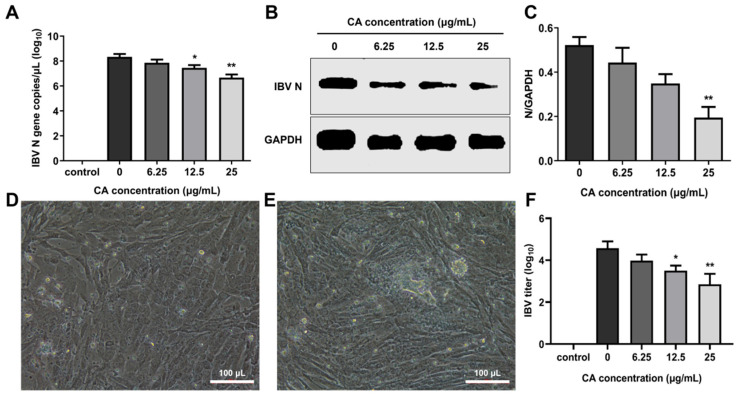
Antiviral effect of CA in vitro. (**A**) CA decreased IBV N gene expression. (**B**) CA decreased IBV N protein expression. (**C**) Gray value analysis of western blotting results. (**D**) The control group cells (20× magnification). (**E**) CPE phenomenon (20× magnification). (**F**) Changes in virus titer in cell supernatant. * indicates *p* < 0.05 and ** indicates *p* < 0.01.

To evaluate the in vitro antiviral effects of CA, CEK cells infected with IBV were administered varying concentrations of CA, and the IBV N gene mRNA and the N protein expression levels were measured at 24 h post-infection (hpi). The results showed that the IBV N gene mRNA and protein expressions decreased in a dose-dependent manner, especially at 25 μg/mL of CA (Figure 5A,B). Additionally, a decrease in viral infectivity was observed in the IBV-infected group treated with 6.25, 12.5, and 25 μg/mL of CA compared to the 0 μg/mL of the CA group. Compared to uninfected cells (Figure 5D), IBV-infected CEK cells exhibited CPE characterized by cell clustering to form syncytia or exfoliated cells adhering to non-exfoliated cells (Figure 5E). The reduction in infectivity was positively correlated with the increasing CA concentrations (Figure 5F). These findings suggest that CA effectively inhibits IBV replication (Figure 5F), as further evidenced by CPE observed in CEK cells (Figure 5E). Collectively, these results indicate that CA can suppress IBV replication in vitro.

### 3.6. CA Inhibits IBV Replication In Vivo

**Figure 6:**
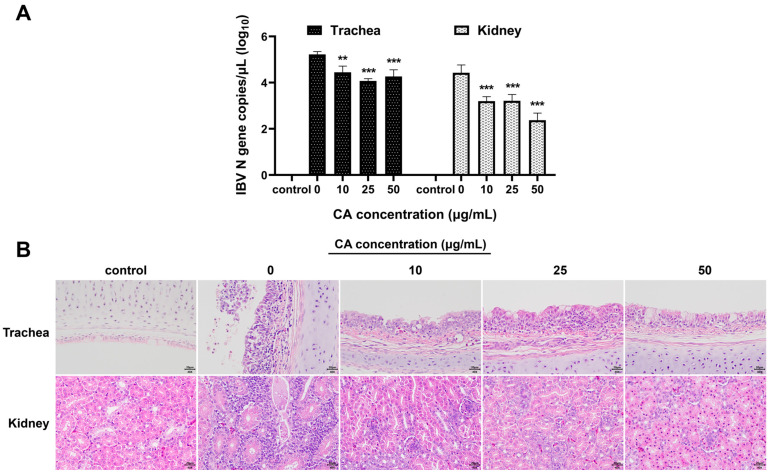
Antiviral effect of CA in vivo. (**A**) CA decreased IBV N gene expression of trachea and kidneys. (**B**) CA affects the histopathological manifestations of trachea and kidneys infected with IBV (Stain H&E). ** indicates *p* < 0.01, and *** indicates *p* < 0.001.

To verify the antiviral effects of CA in vivo, after IBV infection, the experimental chicks were treated with different doses of CA. The viral loads in the trachea and kidneys of experimental chickens were determined, and histopathological changes in tracheal and kidney tissue sections were examined. The qPCR results demonstrated that compared to the blank control group, the viral loads in the trachea and kidneys of IBV-infected chicks significantly increased (Figure 6A). Furthermore, treatment of IBV-infected chicks with different CA doses (10 mg/kg/d, 25 mg/kg/d, and 50 mg/kg/d) showed that CA significantly reduced the expressions of IBV N gene mRNA in the trachea and kidneys of chicks (*p* < 0.01 or *p* < 0.001). The results of the pathological tissue sections revealed that, compared to the blank control group, the renal tubular epithelial cells in the IBV group (with a CA dose of 0 mg/kg/d) exhibited degeneration and infiltration by lymphocytes (Figure 6B). Additionally, the tracheal epithelial cells displayed signs of disintegration and pseudostratified columnar changes, accompanied by extensive inflammatory cell infiltrations in the tracheal mucosa. Different doses of CA (10 mg/kg/d, 25 mg/kg/d, and 50 mg/kg/d) all have certain alleviating effects on tracheal and kidney damage in IBV-infected chicks, and the effects are dose-dependent. Notably, when the CA dose was 50 mg/kg/d, the degree of damage to the tracheal and renal pathological changes was most effectively restored. These findings, as confirmed by qPCR analysis, indicate that CA exerts a significant antiviral effect in vivo.

### 3.7. Docking of CA with IBV N Protein

**Figure 7:**
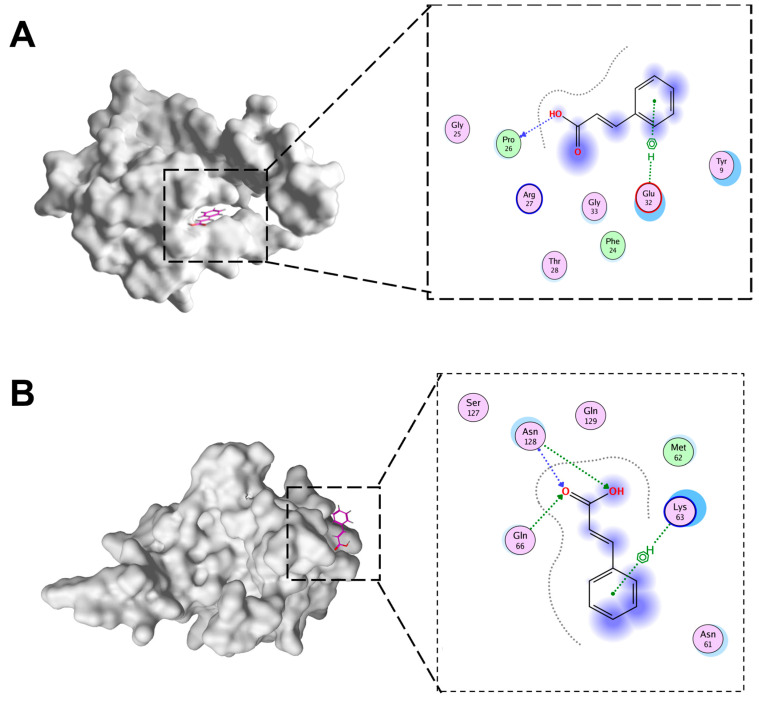
The docking pattern of CA and IBV N proteins. (**A**) CA and IBV N-CTD docking diagram. (**B**) CA and IBV N-NTD docking diagram.

The docking of CA and IBV N protein was conducted in this study. To ascertain which domains of N protein were targeted by CA, potential binding sites were analyzed in detail using MOE to dock CA into the IBV N domain structures (Figure 7A,B). The molecular docking results were evaluated after 10 placement poses for each ligand. Molecular docking revealed binding energies of −4.15 kcal/mol for CTD-CA and −4.37 kcal/mol for NTD-CA. These moderate binding energies align with values reported for fragment-like molecules targeting viral proteins, where functional inhibition occurs despite sub-nanomolar affinity. In addition, the docking of CTD of IBV N protein with CA involved PRO26 and Glu32. The NTD of BV N protein docking with CA involved ASN128, Glu66, and Lys63.

### 3.8. Results of Molecular Docking Validation

**Figure 8:**
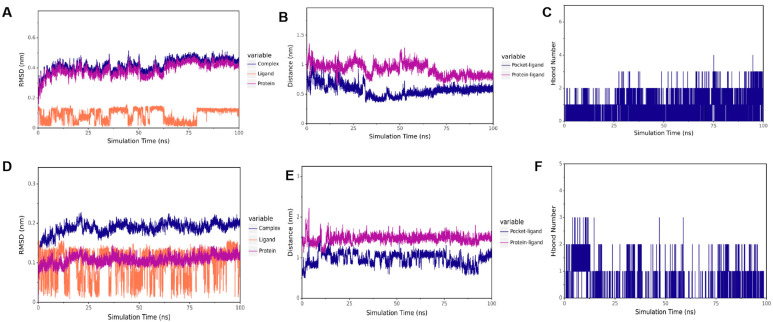
Molecular dynamic of CA and IBV N protein docking complex. (**A**) RMSD of CTD-CA. (**B**) The distance between CA and CTD. (**C**) The number of hydrogen bonds of CTD-CA. (**D**) RMSD of NTD-CA. (**E**) The distance between CA and NTD. (**F**) The number of hydrogen bonds of CA-NTD.

To verify the results of our docking model, we conducted a 100 ns molecular dynamics simulation to evaluate the stability of the receptor and ligand based on the distances between RMSD, CA, and the active bag of the protein, as well as the number of hydrogen bonds. As presented in Figure 8A,D, the results showed that in CTD-CA (IBV N protein) simulation, the RMSD remained stable within 15–60 ns, and in NTD-CA (IBV N protein) simulation, the RMSD remained stable after 75 ns. The results of RMSD when the complex binding was stable are shown in Table 2. As shown in Figure 8B, the distance between CA and the IBV N-CTD protein center, as well as the distance between CA and the initial binding site, remained stable within 15–60 ns. As shown in Figure 8E, distances between CA and the active pocket of IBV N-NTD fluctuated within 0–75 ns but gradually stabilized after 75 ns as the simulation proceeded. To better capture the polar interactions between molecules, we measured the number of hydrogen bonds in molecular dynamics simulations. It can be observed in Figure 8C,F that the number of hydrogen bonds between the two domains of IBV N proteins and CA is relatively small and fluctuates between 0 and 1, indicating that CA can dock with the C and N domains of the IBV N protein, respectively. CA can stably bind to IBV N-CTD at 15–60 ns. After 60 ns, CA leaves IBV N-CTD. CA stably binds to the IBV N-NTD protein after 75 ns. Supplementary Figures S1 and S2 show the full RMSD trajectories of all replicates. Convergence refers to the relatively consistent trends of the three lines in the RMSD chart. The three RMSD plots of the NTD protein show that it has convergence (Figure 8A–C, Supplementary Figures S1A–C and S2A–C). In the RMSD map of the CTD protein, only one map appears to have convergence, while the effects of the other two are not very good (Figure 8D–F, Supplementary Figures S1D–F and S2D–F). Thus, CA stably targets NTD but not CTD.

## 4. Discussion

IBV causes massive economic losses to the poultry industry. Vaccination is still the major measure for the prevention and control of IB [1,2]. However, IBV is prone to mutate, which causes great difficulties in the prevention and control of the disease [3]. In addition to safe and efficient vaccines, broad-spectrum antiviral drugs are urgently needed. Therefore, it is extremely necessary to conduct research on its pathogenic mechanism. At present, the research on pathogenic mechanisms mainly focuses on viral structural proteins and non-structural proteins [24,37], and there are also studies of the host factors dependent on viral replication [38]. However, there are few studies from the perspective of metabolites. Metabolites can best reflect the phenotypic changes in the life activities of an organism. Changes in metabolites can help to understand biological processes and their mechanisms more intuitively and effectively [39]. Therefore, it is highly necessary to conduct metabolomic research on IBV infection.

In this study, serum metabolites in IBV-infected chicks were analyzed using HPLC-MS/MS to characterize the metabolomic profiles. Our study revealed that the metabolic profiles in the sera of IBV-infected chicks were altered, and 143 differentially expressed metabolites were identified, among which, 58 were up-regulated and 85 were down-regulated. Notably, the differential metabolites in sera were mainly related to the phenylalanine pathway. The level of CA, an upstream factor in the phenylalanine pathway, increased following IBV infection. However, our research results are different from those of Kuang et al., who found that the differentially expressed metabolites in the kidneys were positively correlated with Toll-like receptors (TLR7) [11]. Another study showed that the differential metabolites in the bursa of Fabricius after IBV infection are related to the biosynthesis of unsaturated fatty acids, the biosynthesis of amino acids, glycerol phospholipid metabolism, and purine metabolism [12]. This difference might be caused by different viruses, animal species, sampling tissues, and times [40]. In our study, IBV GX-YL5 was used to infect 17-day-old SPF chicks, and serum metabolomics analysis was performed 7 DPI. In the previous studies, IBV SX9 strain was used to infect 28-day-old commercial chickens, and the kidney and bursa of Fabricius metabolomics analyses were performed 10 DPI [11,12]. In our study, the viral loads in the trachea and kidneys of infected chicks showed a downward trend at 7 DPI, but the serum antibody was in a sharp upward trend, indicating that 7 DPI is a key time point reflecting the interaction between IBV and host. Therefore, 7 DPI was chosen for the serum metabolomics analysis. Metabolomics can be used to analyze the metabolic characteristics of diseases, which can reveal the pathological mechanism of diseases, thus providing new strategies for the diagnosis, prevention, and treatment of diseases. At present, many studies use metabolomics to find biomarkers and analyze the pathogenesis of diseases [13]. In our study, a total of 143 differential metabolites were identified involving 20 metabolic pathways, among which, the phenylalanine metabolic pathway changed the most significantly. The expression levels of cinnamic acid (CA) significantly increased in chicks infected with IBV, suggesting that CA metabolite is related to anti-IBV and can serve as potential diagnostic biomarkers for IBV infection in chicks.

Investigating the functional roles of differential metabolites is critical for identifying effective anti-IBV strategies. Metabolomics analysis can also be used to study the prevention and control strategies of diseases. However, there are few studies on the prevention and control strategies of diseases based on metabolomics. At present, there are no metabolomics studies on the prevention and control of IB. Therefore, based on sera metabolomics, we investigated the pathological process of IBV infection in chicks and further studied the prevention and control strategy of IBV. The results showed a marked increase of CA in sera from IBV-infected chicks at 7 DPI, suggesting a potential association between CA and the immune response to viral infection. Previous descriptions showed that CA could target the SARS-CoV-2 nucleocapsid protein to exert its antiviral effect [40]. CA inhibited the Zika virus by inhibiting RNA-dependent RNA polymerase (RdRp) activity [16]. CA inhibits the hepatitis C virus via the induction of oxidative stress [17]. In our study, 7 DPI was a key time point reflecting the interaction between IBV and host. Therefore, we estimated that CA has antiviral effects. This metabolic strategy—specifically HCA-mediated amplification of flavan-3-ol biosynthesis—reflects a conserved defense mechanism observed across biological kingdoms. Similar phenylalanine pathway activation occurs in Zika virus infection, where cinnamic acid derivatives inhibit viral RdRp [41]. Strikingly, this contrasts with SARS-CoV-2’s subversion of tryptophan metabolism for immune evasion via kynurenine upregulation [42], and Klebsiella pneumoniae’s induction of retinoic acid to reinforce mucosal barriers [43]. To further elucidate the role of metabolites in the disease process, we investigated the antiviral effects of metabolite CA at the cellular level. Our findings revealed that CA exhibited non-toxic properties at concentrations below 1036 μg/mL and promoted cell proliferation at lower doses (25 and 50 μg/mL). The qPCR, western blotting, and residual virus infectivity assays demonstrated that CA at 25 μg/mL significantly inhibited IBV N gene and protein expression in vitro, indicating that CA has a significant inhibitory effect on IBV replication in vitro. Which stage of IBV replication is inhibited by CA deserves further study.

To further verify whether CA exerts an antiviral effect on IBV in vivo, 14-day-old SPF chicks were infected with IBV and treated with different concentrations of CA at the same time, and the viral loads and histopathological changes in the trachea and kidney were examined. The results showed that treating IBV-infected chicks with different doses of CA indicated that CA significantly or extremely significantly reduced the expression of IBV N gene mRNA in the trachea and kidneys of chicks. Different doses of CA have certain alleviating effects on tracheal and kidney injuries in IBV-infected chicks, and the effects are dose-dependent. Among them, the CA effects of 25 mg/kg/d and 50 mg/kg/d are better. To sum up, CA also has a significant inhibitory effect on IBV replication in vivo.

As mentioned above, CA can significantly inhibit the replication of IBV both in vivo and in vitro. In order to understand the targets of CA acting on IBV, it was necessary to clarify whether CA targets the structural proteins of IBV through molecular docking and molecular dynamics simulation. The N protein is highly conserved in different IBVs and plays an important role in the viral replication cycle and immune response process [44]. The degree of drug influence on the expression of the IBV N gene or protein has been used to evaluate the inhibitory effect of drugs on IBV replication in many studies [45]. Therefore, we performed the docking of CA and IBV N proteins in this study. Molecular docking shows that the binding energy of CA is −4.15 kcal/mol (N-CTD) and −4.37 kcal/mol (N-NTD), which precisely reflects its role as a fragmented molecule (MW = 148 g/mol). Such energies are common for small metabolites targeting conserved viral domains and do not preclude biological relevance. Precedents include benzopurpurin B binding to SARS-CoV-2 NTD (−4.8 kcal/mol) with antiviral efficacy [46], and nucleozin targeting influenza NP (−4.2 kcal/mol), thereby blocking ribonucleoprotein assembly [47]. This supports our finding that CA’s moderate affinity suffices to disrupt IBV replication via N protein interaction. The results showed that CA could stably bind to the NTD and CTD domains of N protein, indicating that CA can inhibit the replication of IBV by targeting the CTD and NTD of N protein. Interestingly, during the 100 ns molecular dynamics simulation process, CA was stably bound with IBV N-CTD during the period of 15–60 ns, while CA was stably bound with N-NTD after 75 ns. The NTD and CTD of the N protein work together to form the ribonucleoprotein complex (RNP), which is one of the core steps in the viral life cycle. NTD is responsible for the specific binding of RNA, while CTD provides structural support through polymerization [8,9]. As far as we know, this is the first study on the docking of CA and IBV molecules. The docking of CA with other structural and non-structural proteins of IBV is highly worthy of further study.

In summary, the serum metabolites of chicks are significantly altered following IBV infection. The expression of metabolite CA was significantly upregulated, and its levels were closely linked to the phenylalanine metabolic pathway. CA exhibits remarkable antiviral effects in vitro and in vivo. CA exerted antiviral effects by mainly targeting the NTD domain of the N protein of IBV. CA represents a promising candidate for both prophylactic and therapeutic interventions against IBV infection. These findings offer new insights into the development of prevention and treatment strategies for IBV and provide innovative avenues for antiviral drug discovery via metabolic platforms. Our next steps include studying the molecular mechanism by which IBV leads to the disorder of the host’s phenylalanine metabolic pathway, the mechanism by which CA exerts antioxidant stress and anti-inflammatory effects during IBV infection, and the relationship between significantly different metabolites CA, HCA, and BCA and intestinal metabolism.

## Figures and Tables

**Table 1:** Animal grouping and treatment.

Groups	Animals	Treatment	
		D14	D14-D18
Blank control	15	—	—
CA-0	15	IBV challenge	—
CA-10	15	IBV challenge	CA (10 mg/kg/d)
CA-25	15	IBV challenge	CA (25 mg/kg/d)
CA-50	15	IBV challenge	CA (50 mg/kg/d)

**Table 2:** The RMSD results when the two complexes are stably combined.

Metric	NTD-CA	CTD-CA
Complex RMSD	0.4192 ± 0.02097 nm	0.1962 ± 0.009804 nm
Ligand RMSD	0.1131 ± 0.01214 nm	0.1222 ± 0.01125 nm
Protein RMSD	0.3797 ± 0.01158 nm	0.09852 ± 0.003329 nm

## Data Availability

The data presented in this study are openly available in [FigShare], reference number [10.6084/m9.figshare.29134496].

## Supplementary Materials

The following supporting information can be downloaded at https://www.mdpi.com/article/10.3390/microorganisms13071633/s1, Figure S1: Molecular dynamic of CA and IBV N protein docking complex; Figure S2: Molecular dynamic of CA and IBV N protein docking complex.
