# Supplementary figures and images for: Significant Increase of Cinnamic Acid in Metabolites of Chicks Infected with Infectious Bronchitis Virus and Its Remarkable Antiviral Effects In Vitro and In Vivo
Source: Microorganisms. 2025 Jul 10;13(7):1633. doi: 10.3390/microorganisms13071633 (PMC12299430; doi:10.3390/microorganisms13071633)

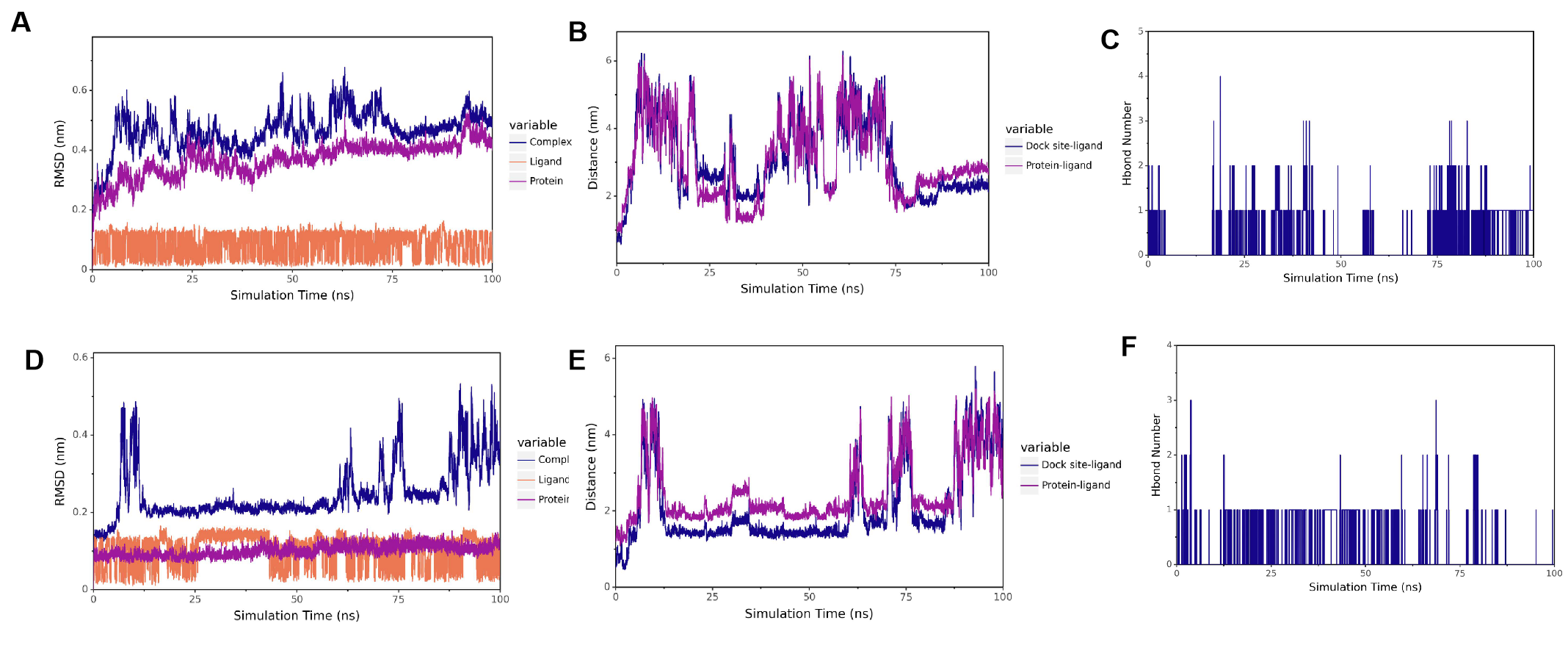

Supplement: Supplementary file 1 [file microorganisms-13-01633-s001.zip › Supplementary Figure S1.tif]

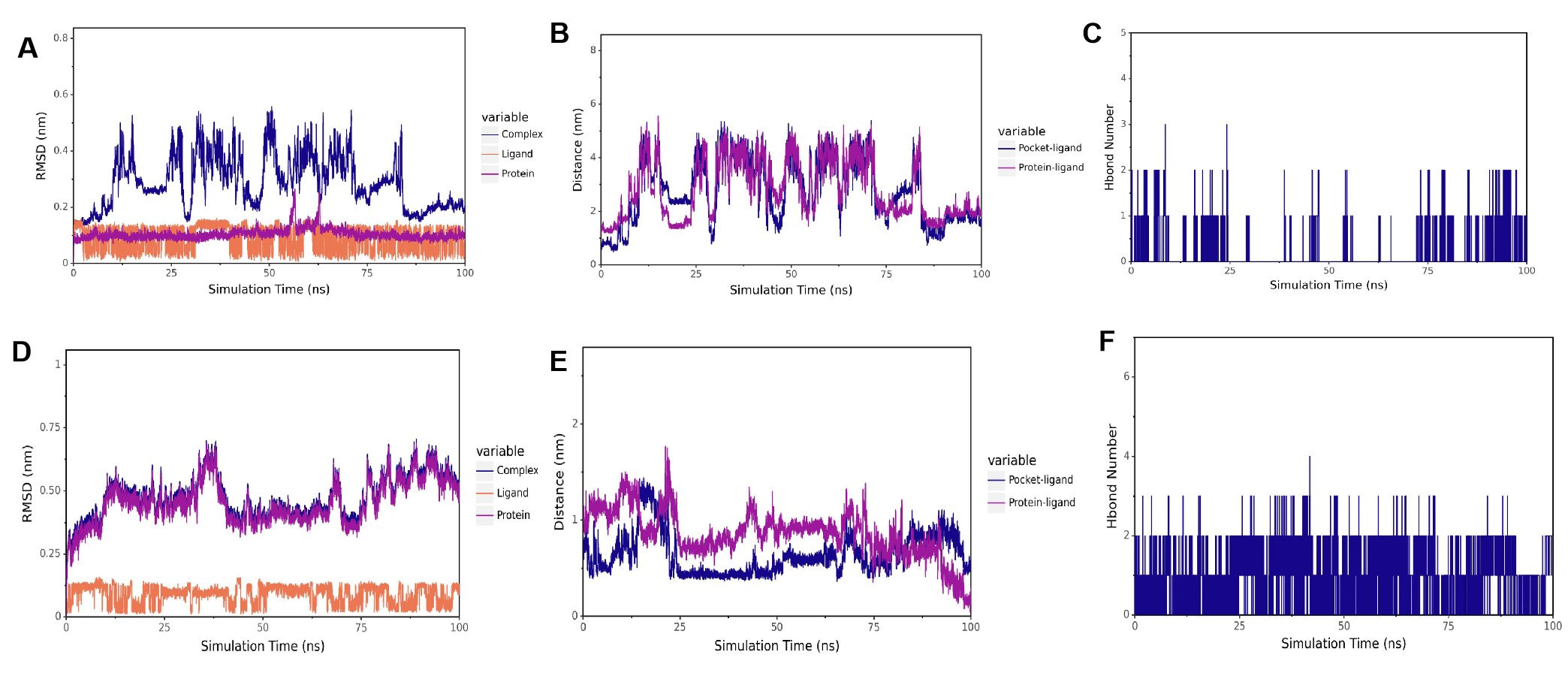

Supplement: Supplementary file 1 [file microorganisms-13-01633-s001.zip › Supplementary Figure S2.tif]
